# Improving Non-Cartesian MRI Reconstruction through Discontinuity Subtraction

**DOI:** 10.1155/IJBI/2006/87092

**Published:** 2007-01-16

**Authors:** Jiayu Song, Qing Huo Liu

**Affiliations:** ^1^ Department of Electrical and Computer Engineering, Duke University, Durham, NC 27708, USA; ^2^ Center for In Vivo Microscopy, Duke University Medical Center, Durham, NC 27710, USA

## Abstract

Non-Cartesian sampling is widely used for fast magnetic resonance imaging (MRI). Accurate and fast image reconstruction from non-Cartesian *k*-space data becomes a challenge and gains a lot of attention. Images provided by conventional direct reconstruction methods usually bear ringing, streaking, and other leakage artifacts caused by discontinuous structures. In this paper, we tackle these problems by analyzing the principal point spread function (PSF) of non-Cartesian reconstruction and propose a leakage reduction reconstruction scheme based on discontinuity subtraction. Data fidelity in *k*-space is enforced during each iteration. Multidimensional nonuniform fast Fourier transform (NUFFT) algorithms are utilized to simulate the *k*-space samples as well as to reconstruct images. The proposed method is compared to the direct reconstruction method on computer-simulated phantoms and physical scans. Non-Cartesian sampling trajectories including 2D spiral, 2D and 3D radial trajectories are studied. The proposed method is found useful on reducing artifacts due to high image discontinuities. It also improves the quality of images reconstructed from undersampled data.


Hindawi Publishing Corporation
International Journal of Biomedical Imaging
Volume 2006, Article ID 87092, Pages 1–9
DOI 10.1155/IJBI/2006/87092

### Improving Non-Cartesian MRI Reconstruction through Discontinuity Subtraction

**Jiayu Song[1, 2] and Qing Huo Liu[1]**

[1] *Department of Electrical and Computer Engineering, Duke University, Durham, NC 27708, USA*
[2] *Center for In Vivo Microscopy, Duke University Medical Center, Durham, NC 27710, USA*

Received 30 April 2006; Revised 7 September 2006; Accepted 8 October 2006

Recommended for Publication by Tiange Zhuang

Non-Cartesian sampling is widely used for fast magnetic resonance imaging (MRI). Accurate and fast image reconstruction from non-Cartesian $k$-space data becomes a challenge and gains a lot of attention. Images provided by conventional direct reconstruction methods usually bear ringing, streaking, and other leakage artifacts caused by discontinuous structures. In this paper, we tackle these problems by analyzing the principal point spread function (PSF) of non-Cartesian reconstruction and propose a leakage reduction reconstruction scheme based on discontinuity subtraction. Data fidelity in $k$-space is enforced during each iteration. Multidimensional nonuniform fast Fourier transform (NUFFT) algorithms are utilized to simulate the $k$-space samples as well as to reconstruct images. The proposed method is compared to the direct reconstruction method on computer-simulated phantoms and physical scans. Non-Cartesian sampling trajectories including 2D spiral, 2D and 3D radial trajectories are studied. The proposed method is found useful on reducing artifacts due to high image discontinuities. It also improves the quality of images reconstructed from undersampled data.

Copyright © 2006 J. Song and Q. H. Liu. This is an open access article distributed under the Creative Commons Attribution License, which permits unrestricted use, distribution, and reproduction in any medium, provided the original work is properly cited.

### 1. INTRODUCTION

Non-Cartesian data sampling, such as radial and spiral encoding, is often performed for fast data acquisition in MRI to capture the rapidly decaying signals. Therefore, non-Cartesian trajectories have gained increased attention in various applications such as functional brain imaging, hyperpolarized gas imaging, contrast-enhanced MR angiography, and cardiac imaging. However, their disadvantages lie primarily in more complicated image reconstruction. The most widely used MRI reconstruction scheme generally consists of three steps: preweighting, gridding by convolving the original data with interpolation kernels, and $d$-dimensional inverse Fourier transform [1]. Conventional gridding methods have used predefined gridding kernel functions, such as triangle, Gaussian, *sinc*, and Kaiser-Bessel functions, and so forth [1, 2].

The recent introduction and development of NUFFT algorithms [3–7] provide an efficient tool to evaluate the nonuniform discrete Fourier transform (NUDFT). The NUFFTs calculate optimal kernels with selected scaling function, so that the interpolation error is minimized in a least-square sense. Some of these NUFFT algorithms have been applied to spiral and radial MRI reconstructions [8, 9] and achieved better accuracy-speed trade off. These NUFFT-based direct reconstructions are referred to as direct reconstruction methods in the rest of the paper.

Unfortunately, NUFFT reconstruction, under the same gridding scheme, provides improvement only on the interpolation accuracies. In fact, image quality is still challenged by $k$-space truncation and density compensation for the non-Cartesian sampling, and as a result, the point spread function (PSF) is not as sharp. For an imaging system, PSF is one of the most important characteristics and ideally should be a single delta function. Lauzon and Rutt [10] studied the "principal PSF," the Fourier transform of the sampling function, for polar (2D radial) sampling trajectory and observed intensity leakage (existence of sidelobes and broadening of the mainlobe). Since the final image is a convolution of the PSF with the true magnetization distribution, the leakage of PSF then results in intensity leakage in the reconstructed image. Furthermore, the intensity of artifacts is proportional to the intensity of the region being convolved. As a result, the artifacts leaked by high intensity regions into their surrounding low intensity regions become most visible. For example, the skull in brain images usually has very high intensity and its leakage into inside of the brain significantly degrades the image quality in various regions of interest.

A straightforward approach to improve the reconstruction result is to sharpen the PSF, which directly corresponds to an optimized selection of density compensation function (DCF). For non-Cartesian sampling, DCF is applied to the raw data to compensate for the sampling density. However, the mathematically ideal DCF [1, 11–13] may not provide highest image quality. Some recent works [14–17] formulate the DCF computation as an optimization problem to emphasize certain PSF characteristics, for example, narrow mainlobe and low sidelobes. Nevertheless, the optimized solutions are rarely close to being ideal and the computational cost of optimization becomes inevitably high when large data sets are processed.

Instead of correcting PSF itself, this paper develops an alternative method with discontinuity subtraction, based on the fact that intensity leakage is most severe at high discontinuities. Here we define "high discontinuity" as the structural region that has high intensity difference (contrast) with the surrounding regions, such as the skull. Similar ideas can be found in some other applications such as computerized tomography [18] and seismic data processing [19]. However, the proposed method does not require any a priori knowledge about the image, which is needed in [18]. The computational cost is slightly higher than direct reconstruction but much lower than iterative methods.

The paper is organized as follows. Section 2 develops multidimensional NUFFTs that are used for reconstruction and $k$-space simulation. These NUFFTs (NUFFT-1 and NUFFT-2) are extensions of Liu and Nguyen's idea of 1D least-square NUFFT [5, 6]. In Section 3, the principal PSF of the direct NUFFT reconstruction method [9] is analyzed and the scheme of the discontinuity subtraction method is described. The proposed method is then compared with the direct NUFFT reconstruction method on both spiral and radial trajectories through computer simulation and physical scan studies in Section 4, followed by discussions in Section 5.

### 2. THEORY

### 2.1. *k-space simulation and reconstruction*

In MRI, the spatially encoded MRI signal $s(\mathbf{k})$ in $k$-space can be modeled as the spatial-frequency response of the distribution of the initial transverse magnetization $M_{xy}$ in spatial domain $\mathbf{x}$. The acquired raw data are therefore Fourier coefficients of the underlying $M_{xy}$ whose image $I_{\mathbf{x}}$ can be reconstructed by taking inverse Fourier transform of the sampled signal in $k$-space. Generally, in $d$-dimension,

$$s(\mathbf{k}(t)) = \int_{\mathbf{x}} M_{xy}(\mathbf{x}) e^{-i2\pi\mathbf{k}(t)\cdot\mathbf{x}} d\mathbf{x}, \quad (1)$$

$$I_{xy}(\mathbf{x}) = \int_{\mathbf{k}} s(\mathbf{k}(t)) e^{i2\pi\mathbf{k}(t)\cdot\mathbf{x}} d\mathbf{k}, \quad (2)$$

where $t$ is the readout time.

For numerical evaluation, the integrands must be truncated and discretized. Note that in (1), $M_{xy}(\mathbf{x})$ usually has a finite support because the object is physically bounded, and thus no truncation is needed. Equation (1) does not need a density compensation either since the image is uniformly sampled. However, (2) would require both truncation and density compensation because of the infinite $k$-space support and nonuniform sampling. Discretizing (1) in Cartesian spatial domain and (2) in non-Cartesian $k$-space gives

$$s(\mathbf{k}_m) \approx \sum_{\mathbf{x}_n} I(\mathbf{x}_n) e^{-i2\pi\mathbf{k}_m\cdot\mathbf{x}_n}, \quad m = 0, 1, \ldots, M-1 \quad (3)$$

which assumes the image voxel size in any $p$th dimension $\Delta x_p = 1$, and

$$I(\mathbf{x}_n) \approx \sum_{m=0}^{M-1} w(\mathbf{k}_m) s(\mathbf{k}_m) e^{i2\pi\mathbf{k}_m\cdot\mathbf{x}_n}, \quad n = 0, 1, \ldots, N-1, \quad (4)$$

where $w(\mathbf{k})$ is the preweighting factor (or DCF). In this work, we use Voronoi DCF [13] for spiral trajectory and the Jacobian DCF for radial trajectory.

Equation (3) is named as "$k$-space simulation formula" that simulates non-Cartesian $k$-space samples from a known image, and (4) as "reconstruction formula" that recovers the image from known non-Cartesian $k$-space samples. Since (4) only involves the inverse Fourier transform once, we refer to this reconstruction as direct reconstruction.

In this work, (3) and (4) are evaluated by the multidimensional NUFFTs, which will be briefly summarized in Section 2.2. Comparison of these NUFFT reconstruction and NUFFT $k$-space signal simulation to conventional gridding methods can be found in detail in our previous work [9]. Again, one should note that (3) provides high-fidelity estimation, while (4) is limited in accuracy and usually consists of leakage artifact, which will be discussed in later sections.

### 2.2. *NUFFT-1 and NUFFT-2*

Multidimensional NUFFT-1 (NUFFT of the first type) and NUFFT-2 (NUFFT of the second type) [9] are developed for fast evaluation of the NUDFTs between the non-Cartesian $k$-space and the Cartesian image space. NUFFT-1 evaluates

$$h_{\mathbf{n}} \triangleq \sum_{m=0}^{M-1} H_m e^{i2\pi\mathbf{v}_m\cdot\mathbf{n}}, \quad \mathbf{n} = \mathcal{L}(N_1) \otimes \mathcal{L}(N_2) \otimes \cdots \otimes \mathcal{L}(N_d), \quad (5)$$

where $\mathcal{L}(N_i) = [-N_i/2, (N_i/2 - 1)]$. It is evaluated by (1) calculating the kernels $\Psi_m$ for each $H_m$; (2) convolving $\Psi_m$ with $H_m$; (3) taking regular FFT to find $h_{\mathbf{n}}$.

Similarly, NUFFT-2 calculates

$$G_m \triangleq \sum_{n_1=-N_1/2}^{N_1/2-1} \cdots \sum_{n_d=-N_d/2}^{N_d/2-1} g_{\mathbf{n}} e^{i2\pi\mathbf{v}_m\cdot\mathbf{n}}, \quad m = 0, 1, \ldots, M-1 \quad (6)$$

with the following procedures: (1) calculate a set of $d$-dimensional kernels $\Psi_m$ with least-square criterion for each of the non-Cartesian samples $G_m$ at $\mathbf{v}_m$; (2) take the $d$-dimensional regular FFT on $g_{\mathbf{n}}$; (3) convolve the FFT results with $\Psi_m$ and find $G_m$.

Take NUFFT-1 as an example. Each kernel $\Psi_m$ is computed as a tensor product of 1D least-square kernels [5, 6] solved by $\psi_m = F^{-1}\alpha(\nu_m)$, where

$$F_{jk} = \begin{cases} N, & j = k, \\ \dfrac{w^{(j-k)N/2} - w^{(k-j)N/2}}{1 - w^{(k-j)}}, & j \neq k, \end{cases} \quad (7)$$

$$\alpha_m = i \sum_{\gamma=-1,1} \frac{\sin\left[(\pi/2\mu)(2m - \gamma - q - 2\{\mu\nu_{m'}\})\right]}{1 - e^{i(\pi/\mu N)(2\{\mu\nu_{m'}\}+q-2m+\gamma)}}, \quad (8)$$

where $\mu$ is the oversampling factor and $q$ is the kernel size. The kernel varies from sample to sample, but it is only dependent on the location of each sample. NUFFT-1 is utilized to evaluate (4) and NUFFT-2 is used for (3). Note when NUFFT-2 is used to evaluate (3), the kernel is in fact the conjugate of $\Psi_m$ due to the negative sign in the exponent. As a result, when NUFFT-1 and NUFFT-2 are used together, the two kernels only need to be calculated once and stored to be called at every NUFFT operation.

Our numerical studies find that the NUFFTs compute (6) and (5) at a computational cost which asymptotically approaches that of a regular FFT with $L_2$ approximation error of $10^{-4}$. In this work, we use $\mu = 2$ and $q = 4$.

### 3. METHODS

### 3.1. The point spread function and intensity leakage

System impulse response, also known as point spread function (PSF), is one of the most important performance measurements of an imaging system. The resulting image can be interpreted as a convolution of the object and the system PSF. If we assume the system is ideal before image reconstruction, that is, the raw data are exact spatial frequency responses at the sampling locations, the system PSF becomes simply the Fourier transform of the sampling function

$$\mathrm{PSF}(\mathbf{x}) = \int_{\mathbf{k}} \mathrm{III}(\mathbf{k}) e^{i2\pi\mathbf{k}\cdot\mathbf{x}} d\mathbf{k} = \sum_{m=0}^{M-1} W(\mathbf{k}) e^{i2\pi\mathbf{k}_m\cdot\mathbf{x}}, \quad (9)$$

where the sampling function

$$\mathrm{III}(\mathbf{k}) = \sum_{m=0}^{M-1} \delta(\mathbf{k} - \mathbf{k}_m) \quad (10)$$

and $W(\mathbf{k})$ is the DCF. We define (9) as principal PSF, to be consistent with the one of the polar (radial) MR imaging system given by [10]. It is shown that the PSF is the DFT of DCF. The desired PSF function should have narrow mainlobe width and low sidelobe level.

In Figure 1, we show PSFs of radial and spiral sampling trajectories. The radial trajectory has 400 rays and each ray is oversampled with 183 points within a $k$-region of $[-0.5, 0.5]$. The spiral trajectory has 16 interleaves and 2048 samples on each interleave. The PSFs are calculated on a $128\times128$ Cartesian grid. Both PSFs, as shown in Figure 1, have significant sidelobes and the mainlobe is broadened. When convolved with the ideal object, these PSFs introduce significant intensity leakages. The caused ringing artifacts are most visible at high intensity discontinuities.

In practice, undersampled radial or spiral acquisitions are widely used by MRI researchers to capture rapid physiological images. In these functional studies, temporal resolution usually trades off spatial resolution, so that undersampling is often used to improve the temporal resolution. For non-Cartesian sampling, wellsampling refers to a sampling scenario whose lowest sampling density still satisfies Nyquist criterion, and any scenarios with lower sampling density than that are considered as undersampling. Figure 2 shows that undersampling introduces replica sidelobes besides the normal leakage. These PSF imperfections will be translated to ringing artifacts and streaking artifacts in the images.

### 3.2. Reconstruction and leakage reduction

To reduce these artifacts, one approach is to optimize DCF for the desired properties of PSF, that is, narrow mainlobe and low sidelobes. However, this approach is quite complicated and minimizing the sidelobes is usually at the cost of widening the mainlobe. Alternatively, we consider the problem from a new perspective and propose a discontinuity subtraction method to reduce the artifacts caused by the high discontinuities. Furthermore, unlike the subtraction method suggested by [18], we use direct reconstruction result to find the discontinuities so that no a priori information about the image is needed. During each iteration thereafter, everything is transformed back to $k$-space as a projection onto convex sets (POCS). Subtraction is applied in $k$-space and the data fidelity is ensured. The detailed procedures of our NUFFT-based leakage reduction method are the following.

(1) Given the non-Cartesian $k$-space data $s_0(\mathbf{k})$, apply (4) to find the estimated image $\hat{I}_0(\mathbf{x})$. Here, gradient edge detection and thresholding segmentation techniques are used for simplicity.
(2) Detect the highest discontinuity $\hat{I}_{d_1}$ in the estimated image with existing edge detection and image segmentation techniques.
(3) Apply (3) to calculate the $k$-space signal $s_{d_1}(\mathbf{k})$ corresponding to $\hat{I}_{d_1}$ and subtract it from the original signal $s_0$.
(4) Reconstruct the updated $k$-space data $s_0$ to get a new smoother image $\hat{I}_0(\mathbf{x})$ without the highest discontinuity.
(5) Repeat (2), (3), and (4) until all the large discontinuities above a threshold are subtracted. Add up the subtracted discontinuities $(I_{d_1}, I_{d_2}, \ldots)$ and the final smooth image $\hat{I}_0$.

A flowchart of the algorithm is shown in Figure 3. Since all the reconstructions and $k$-space simulations are based on a same sampling trajectory, the kernel function used in

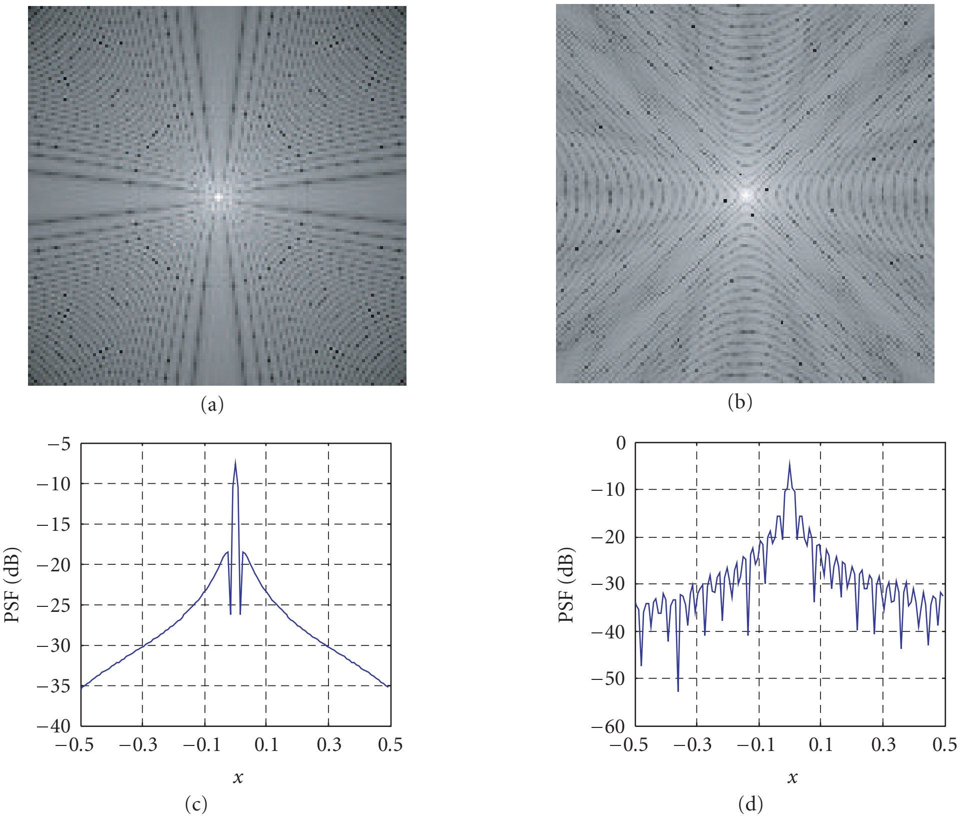

Figure 1: Point spread functions of non-Cartesian sampling functions in dB scale. (a) Radial PSF. (b) Spiral PSF. (c) A center line profile of the radial PSF. (d) A center line profile of the spiral PSF.

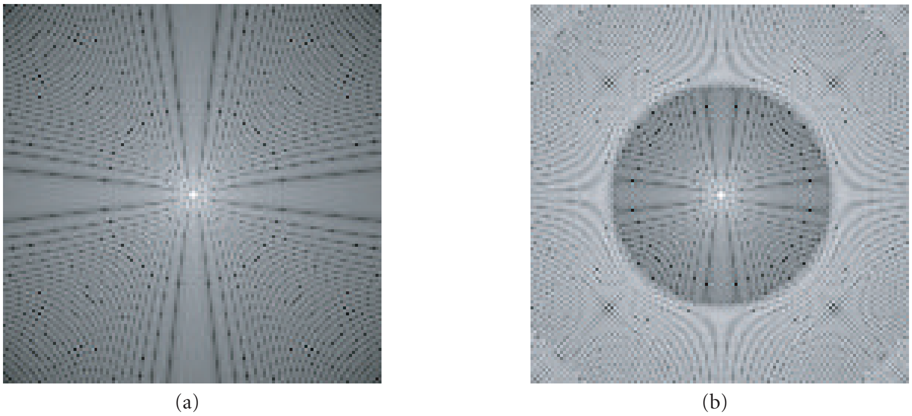

Figure 2: Point spread functions of well-sampled and undersampled radial scans in dB scale. (a) PSF of well-sampled case. (b) PSF of 70% undersampled case.

NUFFT-1 and NUFFT-2 needs to be calculated only once. For example, an image with $m$ high discontinuities will need only one pre-processing, $m + 1$ NUFFT-1 and $m$ NUFFT-2. It takes less than $2m + 1$ times as long as direct reconstruction. Given the number of high discontinuities $m$ in a practical medical image is usually small, the method is faster than most iterative reconstruction methods. For example, $m$ is typically 2-3, which corresponds to 2-3 iterations and takes 5–7 times as long as direct reconstruction.

### 4. RESULTS

### 4.1. Computer simulation study

The Shepp-Logan phantom is utilized here for computer simulation study. It is known as the phantom of "head," which is usually used to simulate brain image with the skull. The non-Cartesian $k$-space signal samples on both radial and spiral trajectories are accurately simulated using (3). The

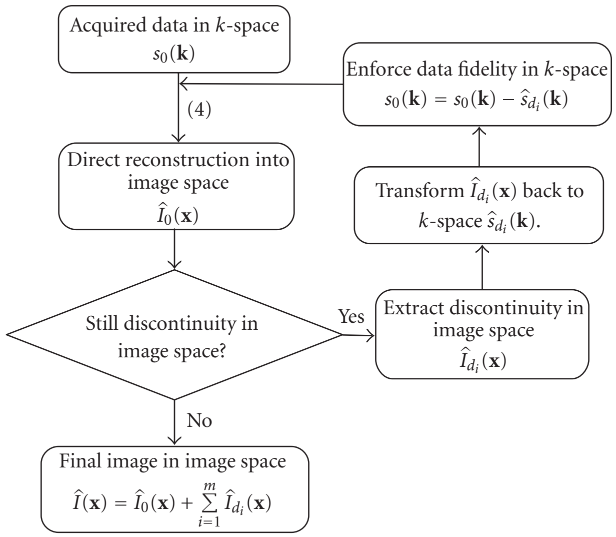

FIGURE 3: Flowchart of the discontinuity subtraction reconstruction algorithm.

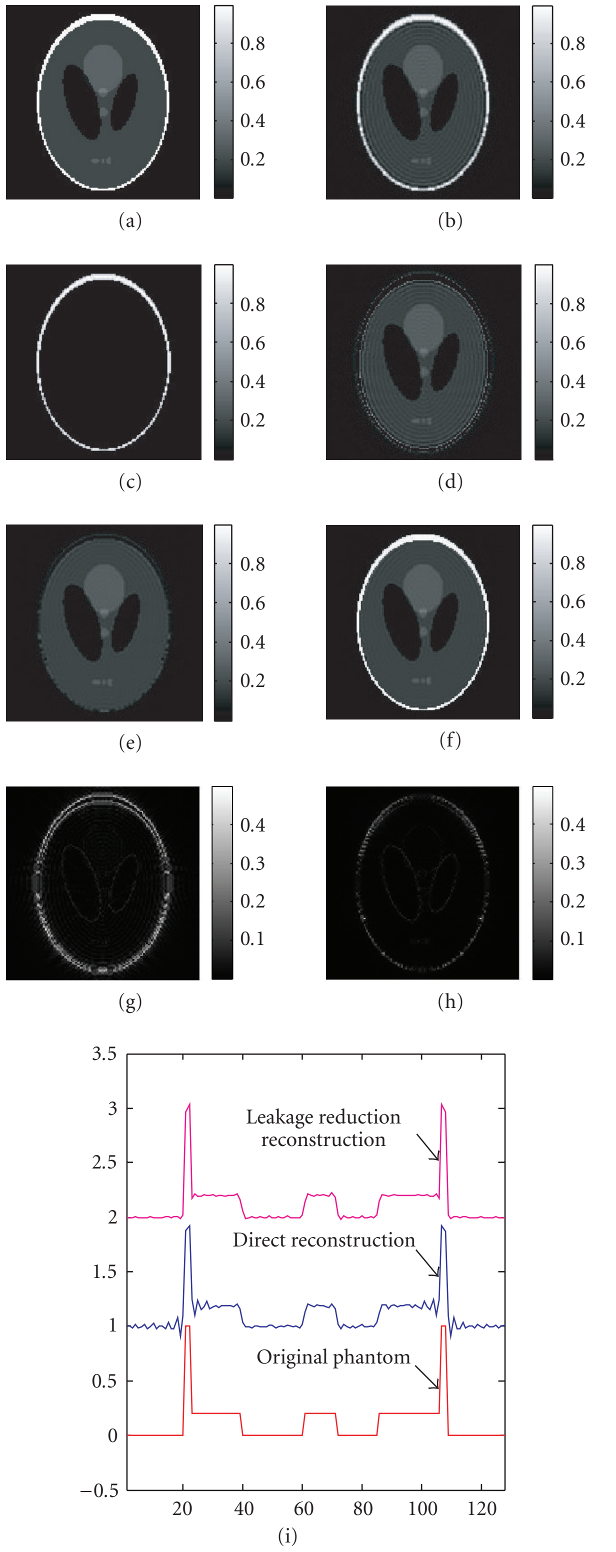

FIGURE 4: Shepp-Logan phantom reconstructed from spiral samples. (a) Original Shepp-Logan phantom image. (b) Direct reconstruction. (c) Highest discontinuity to be subtracted. (d) Remaining image after subtraction of (c). (e) Reconstructed remainder image after one iteration. (f) Final leakage reduction reconstruction. (g) Absolute difference image of direct reconstruction. (h) Absolute difference image of leakage reduction reconstruction. (i) Center line profiles of the reconstructed images.

proposed leakage reduction method is compared with the direct reconstruction on both 2D and 3D phantoms.

Figure 4 shows reconstruction results of a $128 \times 128$ phantom simulated with a 16-interleaved spiral trajectory. Two discontinuities are subtracted during the reconstruction. For illustration purposes, the first high discontinuity image, the corresponding subtraction remainder, and the smoothed remainder are shown in the figure. The direct reconstruction in Figure 4(a) suffers from ringing artifacts caused by the high discontinuity at the "skull." Figure 4(b) shows the highest discontinuity being subtracted, and Figure 4(c) is the remainder. After one POCS iteration, image of Figure 4(c) becomes Figure 4(d). The final reconstructed image by the proposed method is shown in Figure 4(f). Figures 4(g) and 4(h) are error images of Figures 4(a) and 4(f), and horizontal center line profiles are compared in Figure 4(i). The oscillations around the "skull" are visibly mitigated by discontinuity subtraction. Reconstructions of the same $128 \times 128$ phantom simulated with 400 radial rays are shown in Figure 5. Again, the intensity leakages are well improved by discontinuity subtraction.

Table 1 compares the performance of the two reconstruction methods quantitatively and lists the $L_2$ reconstruction errors defined as $e_2 = \|I_{\text{recon}} - I\|_2 / \|I\|_2$, where $I_{\text{recon}}$ is the reconstructed image and $I$ is the original phantom. Both errors decay with increased spatial resolution, but the proposed leakage reduction method performs consistently better than the direct reconstruction method.

In Section 3.1, we discussed the influences of undersampling on the principal PSF. Reconstructing these undersampled data sets becomes more challenging. In Figure 6, we simulated the $k$-space samples of a $128 \times 128$ phantom on only 120 rays (70% undersampled) and applied both direct and leakage reduction methods to reconstruct the image. The streaking artifacts are much less significant when discontinuity subtraction (Figure 6(b)) is applied. The three tiny

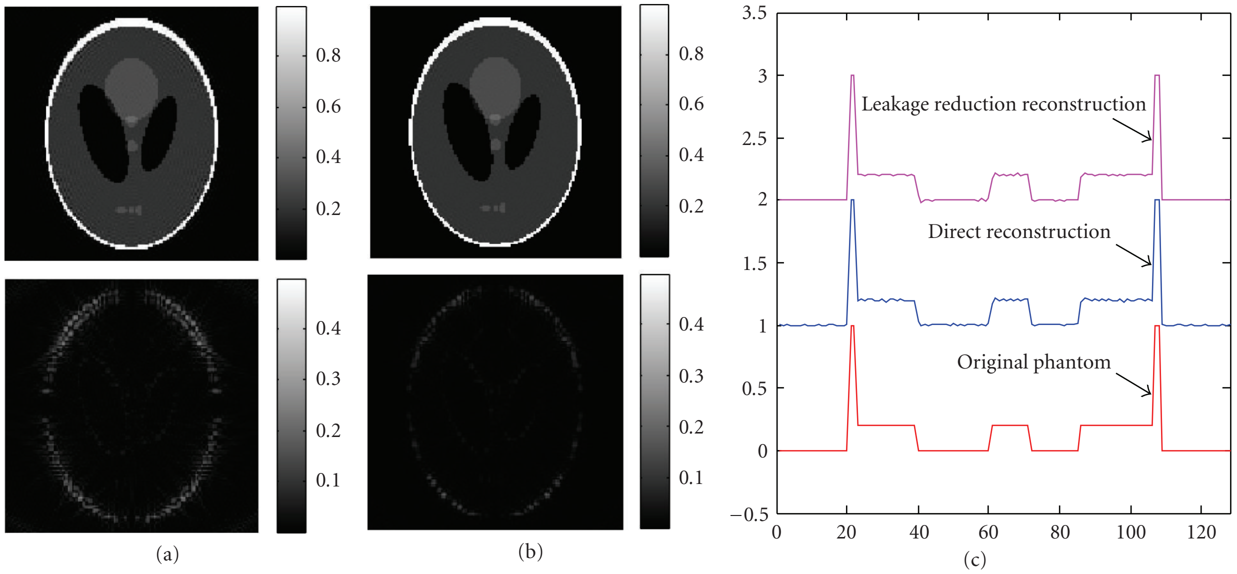

Figure 5: Reconstructed Shepp-Logan phantom images from radial samples and absolute difference images using (a) direct reconstruction, and (b) leakage reduction reconstruction. (c) Center line profiles of the reconstructed images.

Table 1: $L_2$ error comparison of reconstruction results with direct reconstruction ($R_1$) and leakage reduction reconstruction ($R_2$) on spiral and radial phantoms.

| | Spiral | | | Radial | | |
|---|---|---|---|---|---|---|
| | $128^2$ | $256^2$ | $512^2$ | $128^2$ | $256^2$ | $512^2$ |
| $R_1$ | 11.90% | 7.98% | 4.37% | 8.29% | 5.40% | 2.66% |
| $R_2$ | 5.60% | 3.92% | 2.11% | 3.38% | 1.92% | 1.35% |

ellipses near the bottom of the phantom are not resolvable in Figure 6(a) but can be clearly differentiated in Figure 6(b). This is better shown by the line profiles across those three ellipses in Figure 6(c). This suggests an improvement of resolution. In fact, the $L_2$ reconstruction errors are, respectively, 15.91% and 4.33% for Figures 6(a) and 6(b).

A $64^3$ Shepp-Logan phantom is also simulated using 3D isotropic radial trajectory to study the performance of the proposed method in 3D. 12868 rays are simulated to make it a well-sampled case just satisfying the Nyquist sampling rate. Figure 7 shows the three cross planes of the original phantom and reconstructed images using direct reconstruction and leakage reduction reconstruction. The ringing artifacts around the "skull" are significant in Figure 7(b) but are greatly suppressed in Figure 7(c) where discontinuity subtraction is applied. The $L_2$ errors are 25.36% for Figure 7(b) and 11.39% for Figure 7(c), respectively.

Table 2 summarizes the reconstruction processing time of the above experiments using direct method ($T_1$) and the proposed method ($T_2$). The 2D experiments in the table used image size $128^2$ and the 3D case used $64^3$. All of the reconstruction methods are implemented with MATLAB 7.0 on the same computer (CPU: AMD ATHLON 2600+; RAM: 2MB).

### 4.2. *Physical scan study*

Physical MR phantom scans were conducted using spiral and radial samplings as well. A phantom with sharp edges was scanned with a 6-interleaved spiral trajectory. Figure 8 shows the reconstructed images using the two reconstruction methods and center line profiles. Ringing artifacts were generated by the high discontinuities in the phantom structure. Applying the leakage reduction method partially suppressed the artifacts and improved image quality.

Radial scans were also conducted to study the performance of the two methods on undersampled data sets. A cylindrical water tube phantom was scanned by running a 2D radially encoded spin echo sequences on a 2-Tesla Oxford horizontal magnet. We first collected an 800-ray data set to support the Nyquist sampling rate for a 256 × 256 image, then reduced the number of rays to 200 and collected another data set. Direct reconstruction was applied to reconstruct both data sets. It is found that the image of the well-sampled case (Figure 9(a)) looks decent but the one of 3/4 undersampled case in Figure 9(b) has evident-streaking artifacts and low signal-to-noise ratio. These artifacts are mitigated in Figure 9(c) where the proposed leakage reduction reconstruction was applied, and the image quality is visually close to the well-sampled case.

### 5. DISCUSSIONS

The studies described show that the proposed leakage reduction method is helpful on reducing the ringing artifacts near the edges and the streaking artifacts due to undersampling. In this section, we further the discussion on the challenges left to the current algorithm, its applicability, and some potential research directions for improvements.

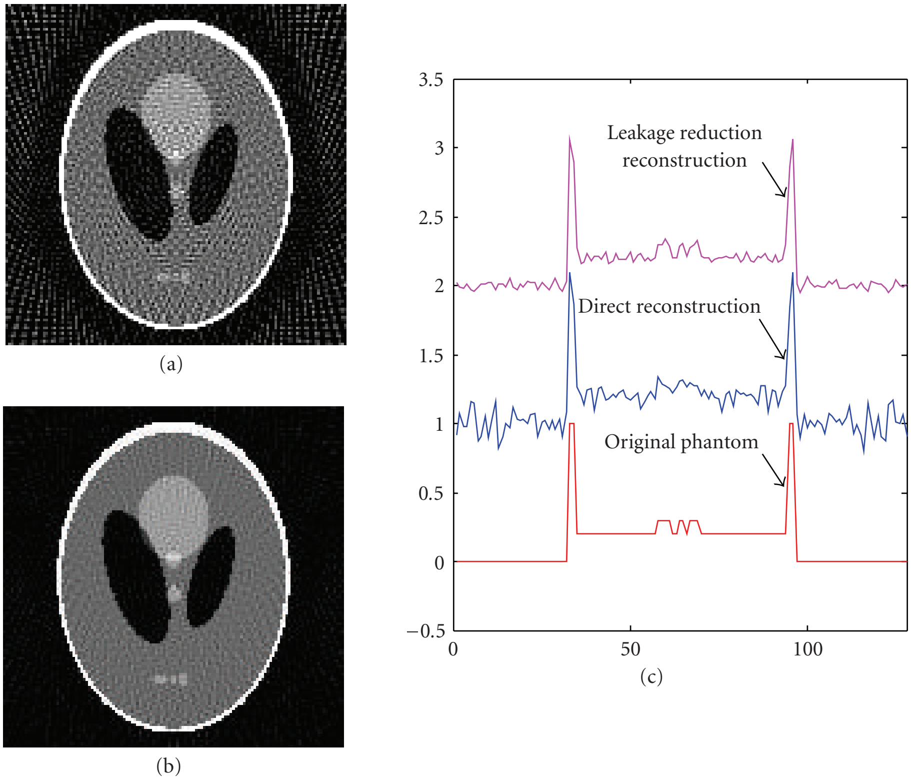

Figure 6: 128 × 128 phantom reconstruction from 70% undersampled radial $k$-space data with (a) direct reconstruction and (b) leakage reduction reconstruction. (c) Line profiles across the three tiny ellipses.

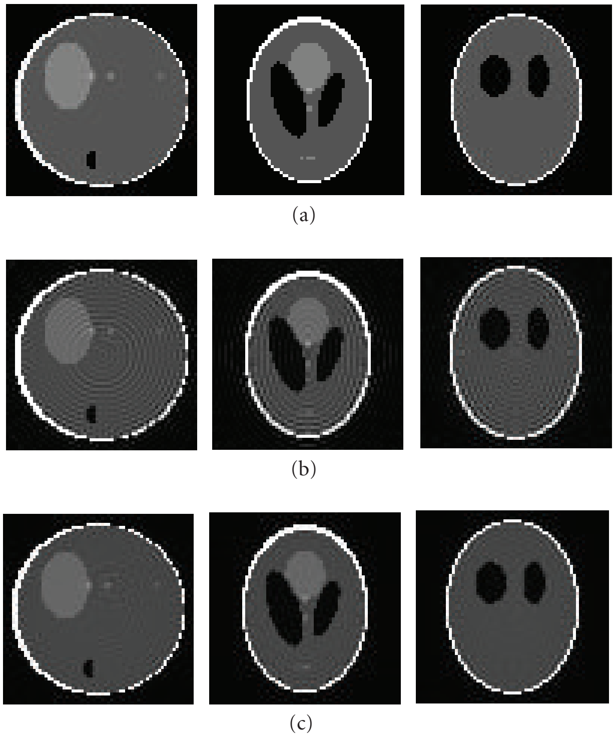

Figure 7: Three central orthogonal cross sections of the 64 × 64 × 64 Shepp-Logan phantom. (a) Original object. (b) Reconstruction from 3D radial $k$-space data with direct reconstruction, and (c) with leakage reduction reconstruction.

Table 2: Processing time comparison of reconstruction results with direct reconstruction ($T_1$) and leakage reduction reconstruction ($T_2$) on 2D and 3D phantoms.

| | 2D spiral | 2D radial | 2D undersampled radial | 3D radial |
|---|---|---|---|---|
| $T_1$ | 1.92 s | 1.59 s | 0.49 s | 59.03 s |
| $T_2$ | 6.32 s | 3.25 s | 0.95 s | 137.09 s |

We found that discontinuity detection, via image segmentation, is quite important to the algorithm. Although the method does not require any a priori knowledge of the object, it should be noted that it does rely on detecting the discontinuity from the direct reconstruction using post-processing techniques. The minimum requirement is that the discontinuities in the direct reconstruction result should be well detectable. It is found that the algorithm may not improve over the direct reconstruction when segmentation does not perform well (such as cases with low discontinuities). We find some cases, for example, those with low signal-to-noise ratio or significantly undersampled $k$-space, extremely challenging. However, it should also be pointed out that the algorithm merely requires a rough segmentation of the current highest discontinuities, and this is usually not as challenging as image segmentation tasks in a normal sense. Second, due to the POCS process that ensures data fidelity in the $k$-space, the algorithm at least should not perform worse than direct reconstruction.

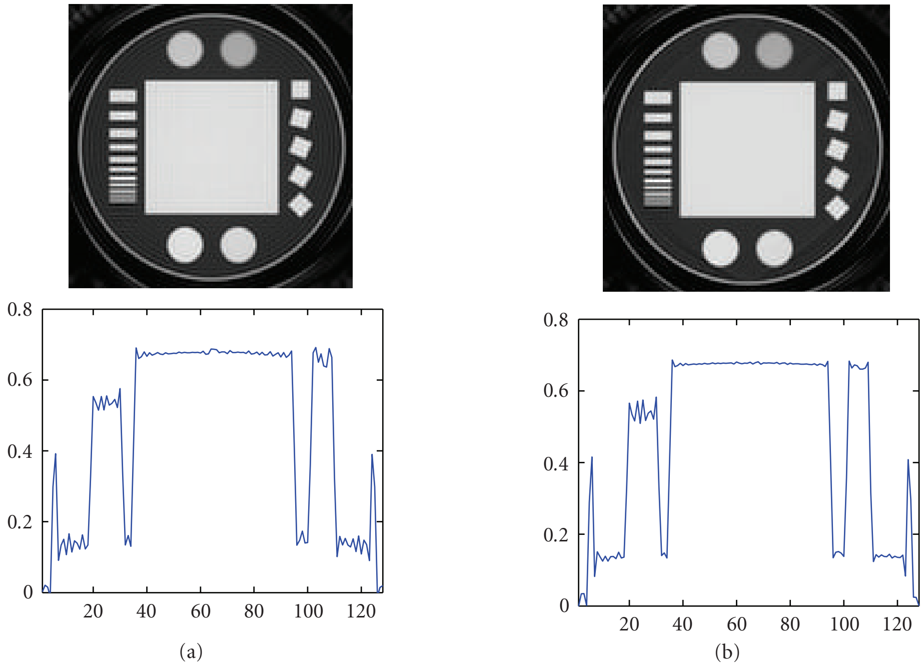

Figure 8: Reconstruction results of a spiral scan through (a) direct reconstruction and (b) leakage reduction reconstruction.

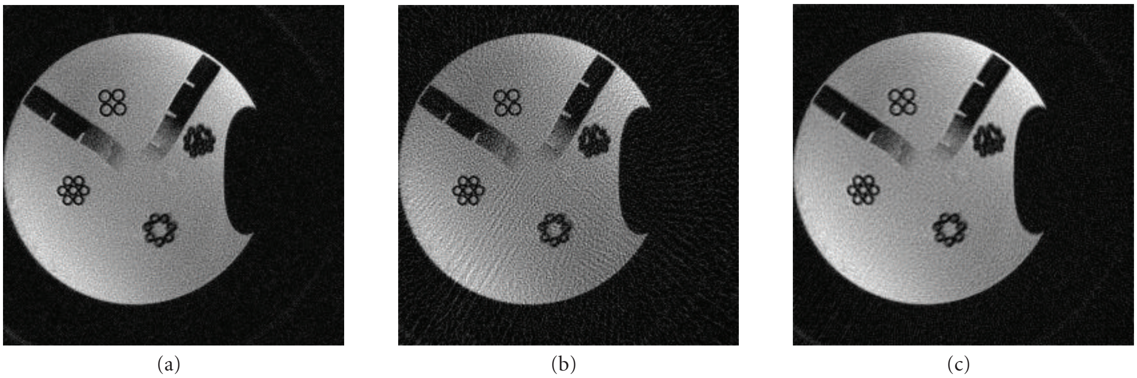

Figure 9: Reconstruction results of well-sampled and undersampled radial scans. (a) 800 rays, direct reconstruction; (b) 200 rays, direct reconstruction; (c) 200 rays, leakage reduction reconstruction.

Given the above challenges, the current algorithm is most applicable to imaging objects that involve high intensity discontinuities, for example, the imaging of brain with skull and cardiac imaging. In these applications, the lower intensity anatomies are of more interest so that the reduction of leakages from the higher anatomies into the low intensity regions becomes very useful.

Based on the analysis, we suggest several research directions. First of all, although segmentation is not the emphasis in the work, there is a large body published work on this topic. We refer the reader to this literature for more effective segmentation methods, and we believe that integrating them in the current framework will result in even more improved results. Second, the proposed method is a good alternative to trade off image quality and computational complexity, but it does not guarantee optimal suppression of artifacts. Some recent works [20, 21] suggested encouraging results using constrained optimization methods that minimize certain edge preserving priors (e.g., total variation or wavelet coefficients) with the constraint of data fidelity. The concept can be integrated into our scheme as well, for example, by updating the image using the gradient of the prior(s) before applying the discontinuity subtraction.

### 6. CONCLUSIONS

A leakage reduction reconstruction method has been proposed for improving non-Cartesian MR image reconstruction. The study explored two major non-Cartesian sampling trajectories, spiral and radial. Studies have shown that principal PSF imperfections cause significant image artifacts when discontinuous structures exist or when the $k$-space signal is undersampled. The proposed method uses discontinuity subtraction and the discontinuity is found in the image space by post-processing techniques. The subtraction is taken in the $k$-space, in order to enforce data fidelity. In this scheme, the multidimensional NUFFTs are exploited for $k$-space simulation and direct image reconstruction. Both computer-simulated Shepp-Logan phantoms and physical scanned data were reconstructed to compare the proposed method with the direct reconstruction method. Results suggested that the proposed method successfully reduced artifacts in images with discontinuities or ones reconstructed from incomplete data.

### ACKNOWLEDGMENTS

The authors would like to thank Dr. John Pauly for providing the spiral scan data. The research was supported by NIH through Grant no. 5R21CA114680. The radial MRI scans were performed at the Duke Center for In Vivo Microscopy, an NCRR/NCI National Biomedical Technology Resource Center (P41 RR005959/R24 CA092656).

### REFERENCES

[1] J. I. Jackson, C. H. Meyer, D. G. Nishimura, and A. Macovski, "Selection of a convolution function for Fourier inversion using gridding," *IEEE Transactions on Medical Imaging*, vol. 10, no. 3, pp. 473–478, 1991.

[2] J. D. O'Sullivan, "A fast sinc function gridding algorithm for Fourier inversion in computer tomography," *IEEE Transactions on Medical Imaging*, vol. 4, no. 4, pp. 200–207, 1985.

[3] A. Dutt and V. Rokhlin, "Fast Fourier transforms for nonequispaced data," *SIAM Journal on Scientific Computing*, vol. 14, no. 6, pp. 1368–1393, 1993.

[4] G. Beylkin, "On the fast Fourier transform of functions with singularities," *Applied and Computational Harmonic Analysis*, vol. 2, no. 4, pp. 363–382, 1995.

[5] Q. H. Liu and N. Nguyen, "An accurate algorithm for nonuniform fast Fourier transforms (NUFFT's)," *IEEE Microwave and Guided Wave Letters*, vol. 8, no. 1, pp. 18–20, 1998.

[6] N. Nguyen and Q. H. Liu, "Regular Fourier matrices and nonuniform fast Fourier transforms," *SIAM Journal of Scientific Computing*, vol. 21, no. 1, pp. 283–293, 1999.

[7] J. A. Fessler and B. P. Sutton, "Nonuniform fast Fourier transforms using min-max interpolation," *IEEE Transactions on Signal Processing*, vol. 51, no. 2, pp. 560–574, 2003.

[8] L. Sha, H. Guo, and A. W. Song, "An improved gridding method for spiral MRI using nonuniform fast Fourier transform," *Journal of Magnetic Resonance*, vol. 162, no. 2, pp. 250–258, 2003.

[9] J. Song, Q. H. Liu, S. L. Gewalt, G. P. Cofer, and G. A. Johnson, "Least square NUFFT methods applied to 2D and 3D radially encoded MR image reconstruction," to appear in *IEEE Transactions on Medical Imaging*.

[10] M. L. Lauzon and B. K. Rutt, "Effects of polar sampling in $k$-space," *Magnetic Resonance in Medicine*, vol. 36, no. 6, pp. 940–949, 1996.

[11] S. J. Norton, "Fast magnetic resonance imaging with simultaneously oscillating and rotating field gradients," *IEEE Transactions on Medical Imaging*, vol. 6, no. 1, pp. 21–31, 1987.

[12] R. D. Hoge, R. K. S. Kwan, and G. B. Pike, "Density compensation functions for spiral MRI," *Magnetic Resonance in Medicine*, vol. 38, no. 1, pp. 117–128, 1997.

[13] V. Rasche, R. Proksa, R. Sinkus, P. Boernert, and H. Eggers, "Resampling of data between arbitrary grids using convolution interpolation," *IEEE Transactions on Medical Imaging*, vol. 18, no. 5, pp. 385–392, 1999.

[14] J. G. Pipe and P. Menon, "Sampling density compensation in MRI: rationale and an iterative numerical solution," *Magnetic Resonance in Medicine*, vol. 41, no. 1, pp. 179–186, 1999.

[15] J. G. Pipe, "Reconstructing MR images from undersampled data: data-weighing considerations," *Magnetic Resonance in Medicine*, vol. 43, no. 6, pp. 867–875, 2000.

[16] H. Sedarat and D. G. Nishimura, "On the optimality of the gridding reconstruction algorithm," *IEEE Transactions on Medical Imaging*, vol. 19, no. 4, pp. 306–317, 2000.

[17] H. Tan and Y. Zheng, "Point spread function optimization for MRI reconstruction," in *Proceedings of the IEEE International Conference on Acoustics, Speech and Signal Processing (ICASSP '05)*, vol. 2, pp. 477–480, Philadelphia, Pa, USA, March 2005.

[18] D. Gottlieb, B. Gustafsson, and P. Forssen, "On the direct Fourier method for computer tomography," *IEEE Transactions on Medical Imaging*, vol. 19, no. 3, pp. 223–232, 2000.

[19] S. Xu, Y. Zhang, D. Pham, and G. Lambaré, "Antileakage Fourier transform for seismic data regularization," *Geophysics*, vol. 70, no. 4, pp. 87–95, 2005.

[20] E. J. Candès, J. Romberg, and T. Tao, "Robust uncertainty principles: exact signal reconstruction from highly incomplete frequency information," *IEEE Transactions on Information Theory*, vol. 52, no. 2, pp. 489–509, 2006.

[21] E. J. Candès and J. Romberg, "Practical signal recovery from random projections," preprint, 2004.

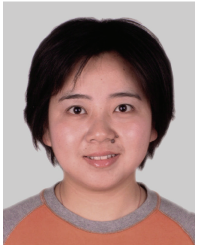

**Jiayu Song** received the B.S. degree in electrical engineering from Tsinghua University, China, in 2003, and the M.S. degree in biomedical engineering from Duke University, in 2005. Since 2003, she has been a Ph.D. student and Research Assistant with electrical and computer engineering at Duke University. Her research interests include image reconstruction algorithms, image processing, and Fourier analysis. She is a Student Member of IEEE.

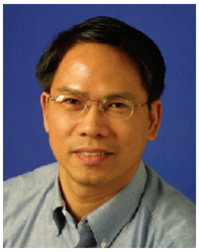

**Qing Huo Liu** is a Professor of electrical and computer engineering at Duke University. He received the Ph.D. degree in electrical engineering from the University of Illinois at Urbana-Champaign, in 1989. His research interests include computational electromagnetics and acoustics, biomedical imaging, inverse problems, geophysical subsurface sensing, electronic packaging, and the simulation of photonic and nanodevices. He is a Fellow of the IEEE and a Fellow of the Acoustical Society of America. Currently, he serves as an Associate Editor for Radio Science, and an Associate Editor for IEEE Transactions on Geoscience and Remote Sensing. He received the 1996 Presidential Early Career Award for Scientists and Engineers (PECASE) from the White House, the 1996 Early Career Research Award from the Environmental Protection Agency, and the 1997 CAREER Award from the National Science Foundation.

